# Genetic architecture of gene expression in the chicken

**DOI:** 10.1186/1471-2164-14-13

**Published:** 2013-01-16

**Authors:** Dragana Stanley, Nathan S Watson-Haigh, Christopher JE Cowled, Robert J Moore

**Affiliations:** 1 CSIRO Animal, Food and Helath Sciences, Australian Animal Health Laboratories, Geelong, VIC, 3220, Australia; 2 Poultry Cooperative Research Centre, PO Box U242, University of New England, Armidale, NSW, 2315, Australia; 3 The Australian Wine Research Institute, Waite Precinct, Adelaide, SA, 5064, Australia; 4 Central Queensland University, Higher Education Division, Bruce Highway, Rockhampton, QLD, 4702, Australia

## Abstract

**Background:**

The annotation of many genomes is limited, with a large proportion of identified genes lacking functional assignments. The construction of gene co-expression networks is a powerful approach that presents a way of integrating information from diverse gene expression datasets into a unified analysis which allows inferences to be drawn about the role of previously uncharacterised genes. Using this approach, we generated a condition-free gene co-expression network for the chicken using data from 1,043 publically available Affymetrix GeneChip Chicken Genome Arrays. This data was generated from a diverse range of experiments, including different tissues and experimental conditions. Our aim was to identify gene co-expression modules and generate a tool to facilitate exploration of the functional chicken genome.

**Results:**

Fifteen modules, containing between 24 and 473 genes, were identified in the condition-free network. Most of the modules showed strong functional enrichment for particular Gene Ontology categories. However, a few showed no enrichment. Transcription factor binding site enrichment was also noted.

**Conclusions:**

We have demonstrated that this chicken gene co-expression network is a useful tool in gene function prediction and the identification of putative novel transcription factors and binding sites. This work highlights the relevance of this methodology for functional prediction in poorly annotated genomes such as the chicken.

## Background

Gene co-expression network analysis has recently emerged as a new data analysis field that presents an opportunity to extract gene interactions from the large number of gene expression datasets available in the ever growing public databases. Expression data from hundreds of unrelated experiments, covering a range of conditions, can be combined into a single analysis. However, in most cases these data sets have only undergone basic differential gene expression data analysis. This approach has failed to capitalise on the abundance of information available in each dataset since analyses are often limited to a small subset of genes which are selected using arbitrary thresholds. This approach is prone to false findings and, in many cases, hard to reproduce [1]. Gene co-expression network analysis is a systems biology approach which complements traditional differential gene expression analysis. Phenotypic variation is controlled at many levels, some of which are independent of transcript abundance. For example, Hudson *et al.*[2] stated that transcription factor (TF) modifications such as reversible phosphorylation and missense mutations can act independently of TF expression levels and that such a process can be overlooked by standard differential gene expression analysis. Hence, instead of exclusively defining differentially expressed genes, the identification of groups of highly co-expressed (CE) genes or gene modules may facilitate the identification of genes under a common regulatory mechanisms by linking upstream sequence motifs with the known binding sites of transcription factors. By combining a high number of experiments into a single robust analysis, it is possible to minimise the effects of variables that can plague individual experiments [1].

Two major types of co-expression networks have emerged: 1) condition-dependant networks and 2) condition-independent, or condition-free, networks. The former, requires careful selection of datasets to cover a single experimental variable, for example, limited to experiments that investigated environmental stresses, such as temperature, pH, oxygen availability etc. The aim being to identifying clusters of highly co-expressed genes (modules) that control the stress responses. This kind of analysis can be extended to yield new gene annotations as in Childs at al. [3], and provide additional insights into the connections between gene expression and the investigated variable. The latter uses data from a number of different tissues, conditions, strains, and other variables. This type of network analysis has the ability to identify genes whose co-expression is independent of experimental variables and stable. This approach is often used to investigate regulatory elements [3]. Advances in module detection algorithms have elevated microarray analysis to much higher levels than simple identification of differentially expressed genes and Gene Ontology (GO) enrichment analysis.

A hub gene, that is a gene which is among the most highly connected within a module, is likely to be tightly involved in the regulatory mechanisms of all those genes with which it is tightly co-expressed. The identification of common sequence motifs in a module and the integration with other biological metadata such as mRNA databases, protein and metabolic networks can add to our understanding of these networks.

In this study we have generated a condition-free co-expression network to shed light on the rather poorly annotated chicken genome through the investigation of clusters of highly co-expressed genes. We have determined putative functions and possible regulatory mechanisms. The network is available on IntegromeDB public database (http://integromedb.org/) under the present manuscript title.

## Results

**Figure 1:**
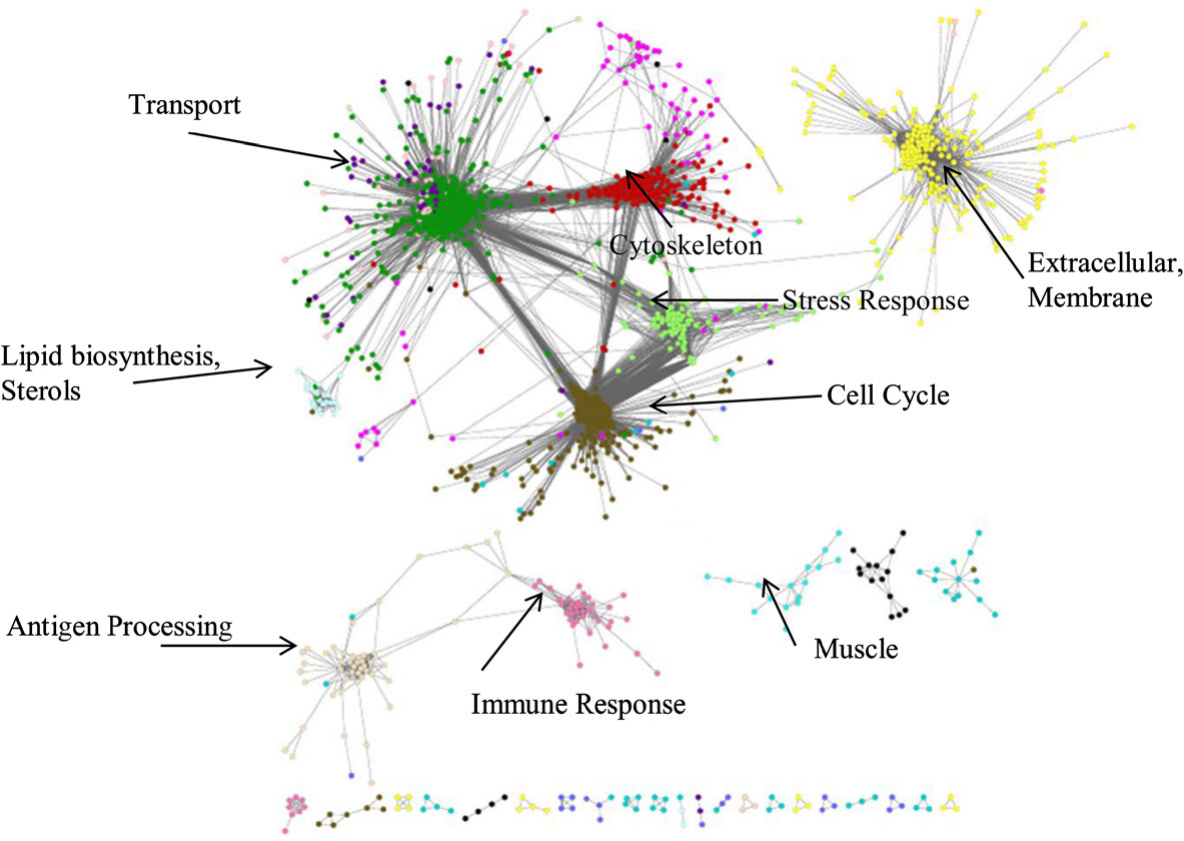
**Cytoscape view of chicken co-expression network.** The nodes are coloured by their module association.

We used 1,043 publically available Affymetrix GeneChip Chicken Genome Array hybridization results (Additional file 1: Table S1) to construct a condition-free, gene co-expression network using the Weighted Gene Correlation Network Analysis (WGCNA) algorithm. We identified 15 clusters of highly co-expressed genes (modules) containing 2,087 (24%) genes from the 8,650 most variable genes used to construct the network. The resulting network was sparse with 48,827 gene connections out of a maximum of approx. 37.4 million pairwise connections. The resulting network is presented in Figure 1.

### Network structure

Most of the 15 modules identified in the co-expression network have a high level of intra-module connectivity (as expected by the definition of a module) and low inter-module connectivity. However, four of the larger modules (1, 2, 4, and 6) possess a large number of inter-module connections and are found to contain genes involved in major cellular functions such as cell cycle, transport, extracellular components, stress response, protein processing, and DNA repair. Two further modules (9 and 12), containing genes enriched for actin cytoskeleton and alpha-catenin binding GO terms, are highly connected to module 1. Such high levels of connectivity between these three modules as well as their closely related functional enrichment, suggest that these three modules could be merged.

We found that modules 10 and 11, enriched for genes annotated with antigen processing and immune response functions respectively, possess genes whose co-expression link the two modules together.

Several modules were found to contain little or no connections to the rest of the network (i.e. stand-alone modules), representing genes whose expression is not linked to any other module either directly or via an intermediary gene. Examples are modules 3 and 15; enriched for genes involved in extracellular matrix and muscle protein. The existence of stand-alone modules does not imply that they are isolated from the biological network, but rather that we do not have all genes present in our network since we limited our analysis to the most highly variable genes.

We found that the topology/structure of the network/modules and biological interpretations were robust to different network construction parameters and analyses (data not shown). We found that increasing the number of genes, from which to construct the network, tended to increase the number of inter-modular connections and thus reduce the number of stand-alone modules. However, the main network topology/structure remained unaffected.

### Biological significance of network modules

The biological significance of the modules was investigated by performing Gene Ontology (GO) enrichment and clustering analysis using DAVID [4]. Most of the 15 modules showed extremely high levels of GO enrichment with GO categories enriched as much as 373 fold and p-values as low as 1.27e-22. This is supported by data given in Additional file 1: Table S1 and summarised in Table 1. One of the DAVID features is Functional Annotation Clustering that places similar GO categories, based on the parent/child GO term associations and the number of the shared genes, into a functional cluster. The GO cluster enrichment score is based on geometric mean of member's p-values and is used to rank their biological significance. DAVID only reports significantly enriched clusters; most enriched clusters have the highest score and lowest p-values. We used this feature to estimate the relationship between the GO terms.

**Table 1:** Modules identified using WGCNA and a most enriched GO category in each module

**Module**	**Module size (genes)**	**% of Genes with functional annotation**	**Top enriched GO category**	**Fold GO enrichment**	**GO enrichment p-value**
1. Green	473	93.23	intracellular transport	3.90	2.75E-07
2. Brown	387	82.95	cell cycle	10.08	1.56E-25
3. Yellow	256	62.89	extracellular matrix part	18.83	3.96E-17
4. Red	227	83.26	cytoskeleton	3.88	1.75E-04
5. Turquoise	103	38.83	organ growth	92.03	4.01E-04
6. Greenyellow	99	77.78	response to stress	7.58	8.30E-04
7. Blue	81	32.10	low annotation		
8. Magenta	67	28.36	low annotation		
9. Purple	58	86.21	actin cytoskeleton	13.87	1.74E-02
10. Tan	58	87.93	antigen processing	69.02	1.46E-13
11. Pink	57	80.70	immune response	19.75	4.01E-07
12. Salmon	39	94.87	alpha-catenin binding	314.76	5.89E-03
13. Black	33	69.70	no GO enrichment		
14. Lightcyan	25	84.00	lipid biosynthetic process	38.52	1.97E-10
15. Cyan	24	91.67	muscle protein	76.39	1.69E-20

Additional file 1: Table S1 contains full tables with all of the GO categories and clusters of GO categories enriched.

Module 2 contained 387 genes involved in a number of cell cycle related biological processes such as cell cycle, cell cycle phase, cell cycle process, M-phase, mitotic cell cycle, nuclear division, mitosis, organelle fission and cell division with p-values ranging from 2.44e^-09^ to 6.99e^-28^ and enriched up to 21 fold. DAVID joined these into a single GO cluster with an enrichment score of 16.8. Similarly, DAVID also identified a second GO cluster containing chromosome, centromere region of chromosome, chromosomal part, centromere, chromosomal protein, cytoskeleton GO terms etc (p-values from 1.45e-^23^ to 1.46e^-5^). All significant GO clusters were involved in crucial processes in the mitotic cell cycle (Additional file 1: Table S1).

Although, module 15 is much smaller in size, with only 24 genes, we still found it to be significantly enriched (p-values from 7.94e^-19^ to 3.92e^-06^) with muscle related GO terms such as: muscle protein, skeletal muscle, myofibril, contractile fiber, sarcomere, cytoskeletal protein binding, muscle contraction, cardiac muscle, heart etc.

Module 14 displayed strong lipid related GO enrichment (p-value of 1.97e^-10^) with 42% of the module’s genes belonging to lipid biosynthetic process (fold GO enrichment of 38.5) while the steroid biosynthesis GO term is enriched 120 fold (p-value of 3.25e^-09^). Module 14’s GO terms were clustered into 4 annotation clusters: 1) sterol metabolic process GO categories; 2) fatty acid metabolism related GOs; 3) membrane biological component and 4) nucleotide related GO categories and binding.

Module 3 showed enrichment (p-value of 3.96e^-17^) for extracellular region GO terms and this accounted for more than 20% of the annotated genes in the module. For example, 12 genes that encode collagen constituents of the extracellular matrix all belong to this module, namely COL1A2, COL2A1, COL3A1, COL5A1, COL5A2, COL6A1, COL6A3, COL8A1, COL12A1, COL16A1, COL24A1 and COL24A1/// LOC424525. The members of this module are all very tightly co-expressed. The annotation clustering of related GOs identified clusters such as extracellular matrix GOs (cluster 1, enrichment score 10.55), basement membrane (cluster 2, enrichment score 8.02), cell adhesion (cluster 3, enrichment score 4.57), collagen (cluster 4, enrichment score 4.34), glycosaminoglycan, heparin and carbohydrate binding (cluster 5, enrichment score 3.4), growth factor binding (cluster 7, enrichment score 2.11) etc. All these clusters of GO categories suggest genes involved in the regulation and maintenance of membrane and extracellular structures.

Two modules, 10 and 11, contained genes of high importance for the immune response. Within module 11, 15.5% of the genes belonged to an immune response GO (p-value 4.01e^-07^ and 19 fold GO enrichment) and includes the genes BLB1, BLB2, TLR2-2, TLR7, TLR16, CD74 and IL18. Other enriched (up to 154 fold) GO categories include Immunoglobulin-like, Toll-Interleukin receptor, positive regulation of immune system process, and antigen processing and presentation of peptide or polysaccharide antigen via MHC class II. Enrichment of similar GO categories was seen in module 10 with antigen processing and presentation (p-value of 1.46e^-13^ and 69 fold enriched), MHC class I protein complex (p-value of 3.49e^-12^ and 119 fold enriched), and immune response (p-value of 4.89e^-12^ and 18.8 fold enrichment).

Module 1 is the largest with 473 genes of which 21 are involved in cell transport (p-value 2.75e^-07^), 60 are involved in nucleotide binding (p-value 1.53e^-04^) and 19 are involved in protein localization (p-value 3.66e^-04^). Based on the GO categories associated with this module (Additional file 1: Table S1) it is possible that this is a control module for the cell metabolism controlling transcription and translation within the cell.

### Major hubs in the chicken co-expression network

We identified 133 hub genes in the network and found that GO assignment and clustering showed overlapping ontology functions. For instance, the hub genes were enriched by up to 103 fold for the following GO clusters: cell cycle, binding (adenyl nucleotide binding, purine nucleoside binding, ATP binding, nucleotide binding, nucleoside binding), chromosome segregation and sister chromatid segregation, cytoskeleton and microtubule organisation, condensed chromosome, microtubule and motor activity and DNA replication. All of this confirms well-known associations of hub genes with expression regulatory mechanisms and allows for speculation into the putative regulatory roles of hub genes which have previously been entirely un-annotated or not annotated with a regulatory role. There were 7 hubs without any functional annotations, 5 of those had Unigene IDs: Gga.1334, Gga.8974, Gga.1245, Gga.13855 and Gga.44105. Running blastn of all available sequences for the 5 Unigene IDs on several databases (GeneBank, EMBL, DDBJ and PDB) we found similarities between Gga.8974 and a number of parafibromin genes from different species with sequence identity up to 99%. Alternative name of parafibromin is Cell division cycle protein 73 homolog and it is involved transcriptional and post-transcriptional control as reported on UniPort database. Unigene Gga.1245 showed highest (97%) sequence identity to ubiquitin-conjugating enzyme E2 from *Meleagris gallopavo* (wild turkey) and also from rabbit, Guinea pig, horse, dog, rat etc. Remaining Unigene IDs had no blast hits.

The top three hub genes, in order of connectivity, in the chicken co-expression network are: 1) RING finger protein 4 (RNF4) with connections to a total of 468 other genes; 2) importin 5 (IPO5) with 463 connections; 3) splicing factor 5a (SRSF5A) with 453 connections. In addition, we also found hub genes which were un-annotated. The most connected of these un-annotated hub genes is Gga.1334 with 268 connections to other genes. Such highly connected hub genes should be investigated more closely as they are likely to have key roles in regulating gene expression in the chicken.

### Regulatory elements associated with network modules

In order to identify possible known transcription factor binding sites (TFBS) statistically overrepresented among the genes from each module, the command line version of the Clover software [5] and JASPER CORE database [6] were used. The binding motifs were considered significantly overrepresented in a module if Clover p-value was lower than 0.01. The results are summarised in the Table 2.

**Table 2:** Summary of the Clover analysis of statistically overrepresented (p<0.01) transcription factor binding sites based on Jaspar Core database

**Module**	**Overrepresented TF binding sites**
1	NHP6B, hb, Pax4, br_Z1, br_Z4, SP1, SFL1, NHP6A, id1, NFATC2
2	hb, Pax4, br_Z1, br_Z4, SFL1, NHP6A, id1, NFATC2, Dof2, HCM1
3	hb, Pax4, NFATC2, id1, br_Z4, Myf, Dof2, SFL1, SOK2, CUP2
4	hb, AZF1, br_Z1, br_Z4, id1, SFL1, NFATC2, HCM1, CUP2, SMP1
5	HMG-I/Y, Pax4, hb, br_Z1, Foxd3, SFL1, br_Z4, id1, NHP6A, NFATC2
6	CUP2, slp1, pan, PEND, Gfi, GABPA, NFYA, mirr, ARID3A, EDS1
7	AZF1, Pax4, br_Z1, id1, CUP2, D, Dof2, PHD1, MNB1A, Ubx
8	br_Z3, NFYA, HAP3, HAP5, Gfi, CG34031, TBP, pan, ARR1, CG11617
9	SFL1, id1, NFATC2, CUP2, Myf, SMP1, abi4, Dof2, br_Z3, SPIB
10	IRF1, IRF2, Myf, CUP2, NFATC2, Dof2, SOK2, Sox2, NHLH1, MNB1A
11	SPI1, Myf, MZF1_1-4, SOK2, EBF1, PHD1, RUNX1, achi, vis, ELF5
12	SFL1, id1, Dof2, RME1, ELF5, PEND, MGA1, MNB1A, Gata1, SOX10
13	PHD1, GSM1, MGA1, Ar, Lim1, abd-A, al, INO4, CG11294, CG32105
14	NFYA, HAP5, Myf, NFATC2, PEND, HAP4, TBP, slp1, Dof2, cad
15	Myf, PHD1, AGL3, MNB1A, kni, YAP5, ECM23, GAT4, RLM1, MAC1
HUBS	hb, NHP6B, br_Z1, Pax4, br_Z4, NHP6A, SFL1, id1, NFATC2, HCM1

The table shows top 10 overrepresented motifs, complete table is provided in Additional file 1: Table S1. The motif names and capitalisation are as they appear in Jaspar database.

### Motif discovery associated with network modules

The MEME software suite [7] was used to identify novel upstream motifs which might account for the co-expression/regulation of gene expression of genes within each module.

**Figure 2:**
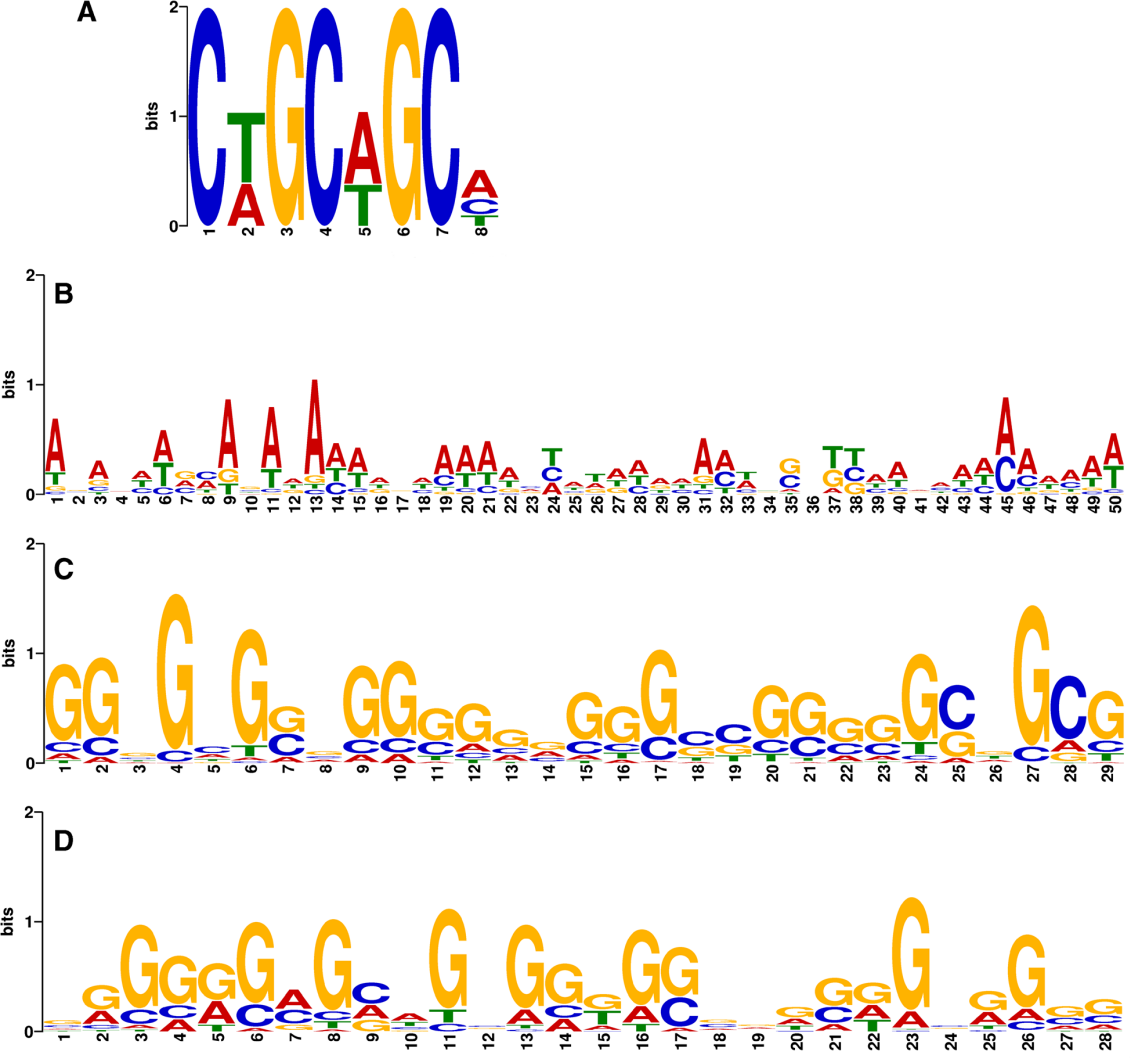
**Novel motifs predicted using MEME software.** Motif **A** was predicted using upstream sequences from module 11, **B** and **C** from module 14 and **D** from the module 15.

When module 11 genes were inspected for the presence of cis motifs, a very ordered structure in the upstream region of 30 un-annotated genes was observed. Closer inspection of these genes using Ensembl and the chicken genome viewer (WASHUC2), shows them to be annotated only as novel genes and “protein coding Ensembl ID”. All of these genes without functional annotations show a surprising level of similarity: Ensembl gene homologue search indicated that they are all homologous to immunoglobulin-like receptors CHIR-A2, CHIR-AB3, CHIR-AB-600, CHIR-AB3, CHIR-B1, CHIR-B2, CHIR-B3, CHIR-B4, CHIR-B5 and CHIR-B6. This confirms that sequence homology continues into the upstream region which is unusually enriched in cis-elements. However, closer gene by gene inspection showed that one Affymetrix feature, Gga.17679.1.S1_s_at, was mapped to 17 Ensembl Gene IDs from the 30 un-annotated genes above, indicating again high sequence homology between these genes that allowed binding to the same array probe. Considering that the remaining well annotated genes from module 11 are significantly enriched in immune response (p-value 4.01e^-07^), the fact that the un-annotated genes share sequence homology with a group of immunoglobulin-like receptors confirms the selectivity and validity of the network modules. The analysis was also repeated using 1 Ensembl Gene ID per one array feature. We identified a novel motif overrepresented with an e-value of 4.1e^-14^, present in 80% of inspected module 11 sequences with 90 sites (Figure 2A). Using the Gene Ontology for Motifs (GOMO) algorithm we found the motif was overrepresented in the upstream regions of genes from GO category “immune system process” (p-value of 1.69e^-05^) and “immune response” (p-value 5.08e^-04^).

Module 14 contains genes from lipid biosynthesis and sterol related GOs; we identified a novel motif (Figure 2B) that is overrepresented (p-value of 2.54e^-05^) in calcium-dependent phospholipid binding GO and another motif (Figure 2C) overrepresented (p-value of 2.54e^-04^) in genes that make up the regulation of lipid kinase activity GO.

The motif that showed the most significant GO association (p-values 8.479e^-06^ to 5.935e^-04^) was detected in muscle related module 15. This motif (Figure 2D) was found to be overrepresented in cis regions of genes annotated with the following GO categories: muscle organ development, muscle cell differentiation, muscle tissue development, striated muscle tissue development, skeletal muscle tissue development, skeletal muscle organ development, regulation of smooth muscle cell migration, muscle fiber development, skeletal muscle fiber development, muscle organ morphogenesis, cardiac muscle tissue morphogenesis, muscle tissue morphogenesis, regulation of skeletal muscle fibre development, striated muscle thin filament, and regulation of muscle cell differentiation. This motif was enriched within the module with an e-value of 2.50e^-33^. A number of other novel motifs were discovered as significantly enriched in different modules; however, the extent of the data far exceeds the scope of this manuscript and allocations for supplementary data. The complete motif analysis can be provided on request to the corresponding author.

## Discussion

The data published in the co-expression network area has clearly confirmed that highly co-expressed genes are enriched for certain cellular functions. One of the major breakthroughs in our understanding of cellular networks was finding that cellular networks display scale-free topology [8,9]. Properties of scale-free networks are highly dependent on a small number of hubs; the nodes with disproportionally high number of connections. It has been demonstrated, using yeast co-expression networks, confirmed with knockout strain studies, that these network hubs are mostly essential genes [8,9]. This organisation of cellular networks shows a surprising degree of tolerance to random errors/perturbation [9,10]. The high number of poorly connected nodes assures that most faulty nodes are quickly by-passed. Even the loss of up to 80% of the nodes will not destroy a scale-free network. However, if such a node happens to be one of the relatively small number of hubs, whether targeted or by chance, the network topology will be seriously affected and will result in network failure [11]. The present network identified a number of key genes in control of chicken gene expression. We found hub genes which were un-annotated, the most highly connected of these is Gga.1334. Such highly connected hub genes should be investigated more closely as they are likely to have key roles in regulating gene expression in the chicken.

The network presented here shows that gene expression in the chicken genome is highly organised and is regulated in concert across a range of tissues and variables. It was previously shown that this “guilt by association” heuristic is universal and preserved beyond organism boundaries [12,13] and that transcriptional control is overwhelmingly modular and appropriate for characterizing gene functions based on module assignment [14]. A human co-expression network, cited by over 300 manuscripts, generated by Lee at al., [15] using 60 human public datasets and over 3000 arrays also shows modularity through the hierarchical clustering and cluster enrichment in certain biological functions. The module functional association of this human network is very comparable with the present chicken network. Both networks contain modules or sub-modules enriched in cell cycle, transcription regulation, immune response, MHCII, transcription, RNA processing, metal binding and cytoskeleton. This is not surprising considering the genome homology between the human and chicken. It also confirms that, when using condition-free networks across a large number of arrays, tissues and conditions, the modules are likely to be associated with universal and essential cellular processes shared across organisms.

It has been shown that multiple shared transcription factor binding sites are necessary for co-expression to occur and that there is a positive correlation in sequence similarity and co-expression [16]. There were a total of 154 TFBS of known experimentally proven transcription factors, significantly overrepresented across the modules. Some of TFBS were overrepresented in most of the modules. Based on the fact that network modules are associated with major cellular processes, transcription factors acting on these TFBS are expected to be of universal significance, independent of external variables, involved in control of some of the major cellular processes.

In modules enriched in more specialised GO categories the TFBSs matched the module assignment. Module 15 was enriched in muscle related GOs such as muscle protein and skeletal muscle. This module had Myf, known to be a muscle-specific transcription factor [17], as the top scoring transcription factor (p-value 0). The top scoring transcription factor in module 11, involved in immune response, was SPI1 also known as PU-1 (p-value 0). SPI1 is known to activate gene expression during myeloid and B-lymphoid cell development [18] and controls macrophage differentiation [19]. In module 10, controlling antigen processing and immune response, the top scoring transcription factors (p-value 0) were IRF1 and IRF2. Both are members of interferon regulatory transcription factor (IRF) family. This provides confidence that novel and un-annotated transcription factors found to be linked to specific GO categories, are involved in the regulation of gene expression within a module. De novo cis element searching also provided additional information confirming exclusive immune response assignment of module 11. Novel motifs found in this analysis can be further investigated and matched to aid in the discovery of novel transcription factors.

## Conclusions

The chicken co-expression network is a useful tool for generating gene function predictions, especially since the chicken genome is relatively poorly annotated. The presented network points to important and essential genes (hubs), novel transcription factors and their binding sites, and predicts likely functional roles of a large number of currently un-annotated chicken genes.

## Methods

### Datasets used

We selected data from 1,043 Affymetrix GeneChip Chicken Genome Arrays (platform number GPL3213), representing 67 different experiments from, ArrayExpress and the Gene Expression Omnibus (GEO). This data covers a wide range of chicken tissues, environmental and health conditions, information on all of the experiments and corresponding arrays is provided in Additional file 1: Table S1. This microarray provides expression data on 32,773 chicken transcripts from over 28,000 genes and 684 transcripts from 17 avian viruses.

Sequence information used in feature selection were derived from GenBank®, UniGene (Build 18; 15 May 2004), and Ensembl (version 1, released May 2004). Affymetrix annotations were downloaded July 2011 and were last updated in May 2006.

### Dataset pre-processing

CEL files were downloaded for the 1,043 microarrays and a series of pre-processing steps were performed in order to normalise, filter and remove batch-effects. Firstly, data was normalised using RMA background subtraction and quantile normalised using the Affy package [19] in Bioconductor. We then removed batch effects using the nonparametric CombatR algorithm [20] and then retained only the most variable genes, based on standard deviation of each gene, as suggested by Hahne and Huber [21], using GeneFilter [20] R package. A total of 8,650 genes were identified as most variable and hence suitable for inclusion in the network building process.

We used the WGCNA algorithm [22,23] to identify 15 condition independent modules of highly co-expressed genes. A total of 8,650 genes were identified as most variable and hence suitable for inclusion in the network building process. Out of the 8,650 features, 2,087 were assigned to co-expressed modules to build a network with 48,827 gene connections. The resulting network is presented in Figure 1.

### Network construction and module detection

We took the 8,650 genes which were retained after the pre-processing steps to construct a gene co-expression network using the WGCNA R package [22,23]. The adjacency matrix was calculated using the absolute Pearson correlation coefficient raised to the power β. The coefficient of β was 4 and was selected based on the scale-free topology criterion which aims to balance scale independence and mean network connectivity [22,23].

The Topological Overlap Measure (TOM) was calculated for performing module detection. Module detection was done using the static tree cutting algorithm [22,23] on the TOM dissimilarity measure using a minimum module size of 25 nodes/genes. All of the remaining arguments were set as recommended in package vignettes.

The co-expression network was visualised using Cytoscape v 2.8.0 [24] and analysed using the NetworkAnalyser Cytoscape plugin.

### Hub gene detection

Hub genes are defined as those genes in the network that are among the most highly connected. They are important nodes in the network as they provide it with structure and are an inherent feature of scale-free networks. We detected hub genes using NetworkAnalyser Cytoscape plugin.

### Biological relevance of modules and hub genes

Modules and hub genes were analysed in a variety of ways in order to ascertain their biological relevance. Firstly, GO enrichment and GO clustering of the genes within each module was performed using DAVID [4]. Unless otherwise stated, p-values are multiple-test corrected using Bonferoni correction. Secondly, we looked at whether there were any significantly overrepresented sequence motifs, using MEME [7], in the CIS regions of the genes within each module and using all suggested default settings unless stated otherwise. Thirdly, we looked at whether known transcription factor binding motifs from the Jaspar database [6] were overrepresented (p<0.01) in the CIS regions of the genes within each module. The overrepresentation of known transcription factor binding motifs was calculated using Clover [5] with 2 backgrounds: 2,000 bp upstream of orthologues on human and mouse genomes.

Unless stated otherwise, we define CIS regions to our module genes as being 200 bp downstream and 1,000 bp upstream of the start of exon 1. The sequence data for these motif analyses was obtained via Toucan [25,26], from the Ensembl database. Sequences corresponding to genes on reverse strand were reverse complimented.

The putative roles of overrepresented sequence motifs, found by MEME, were identified by linking GO terms in upstream regions of orthologous genes in human, mouse, dog horse and rat using MEME’s GOMO algorithm. This would indicate the possible role of novel motifs in the regulation of gene expression for a specific GO category.

## Competing interests

The authors declare that there are no competing interests.

## Authors’ contributions

DS was responsible for project conceptualization, did the complete data analysis and wrote the first draft of the manuscript, NWH and CJEC provided advice and help with data analysis, RJM supervised the project and contributed to the data interpretation and writing of the manuscript. All authors approved and contributed towards the final version of the manuscript.

## Supplementary Material

Additional file 1: Table S1 The file contains a number of tables organised in sheets. The overview of the datasets used in network construction, GO categories enriched in each of the modules and Clover TFBS and MEME analysis. Click here for file
